# The Influence of Tropical Climates, More Especially the Climate of India, on European Constitutions; the Principal Effects and Diseases Thereby Induced; Their Prevention or Removal; and the Means of Preserving Health in Hot Climates, Rendered Obvious to Europeans of Every Capacity. An Essay

**Published:** 1813-09

**Authors:** 


*The Influence of Tropical Climates, more especially the Climate of India, on European Constitutions; the principal Effects and Diseases thereby induced; their Prevention or Removal; and the Means of preserving Health in Hot Climates, rendered obvious to Europeans of every Capacity.*

An

*An Essay, by* James Johnson, Esq. Surgeon in the Royal Navy. 1 vol. 8vo. pp. 536. London. Stockdale, Pall Mall. 1813.

Every publication which results from personal observation in tropical climates, and which points out the dangers or mitigates the diseases to which our countrymen are exposed in those regions, demands our attention, and has a just claim to our earliest notice.

The work before us is ushered into the world under the auspices of Dr. Harness and Dr. Curry, to the former of whom it is addressed, by permission, in a manly and independent dedication. The preface informs us that "it has not been committed to the press till after fifteen years of observation and experience in a vast variety of climates, and with some *unusual sources of information* on the subject which it more particularly embraces." Mr. Johnson here intimates, what indeed the whole of the work amply confirms, that he disdains to copy his predecessors in this walk, from whom he dissents without ceremony, on numerous occasions, "determined (he says) never to succumb to any doctrines or opinions, whatever might be their authority, whenever they clashed with the evidences of his own senses." To this we do not object, and have only to say, O si sic omnia! Mr. J. here also makes a remark which has often suggested itself to us, namely, how singular it is "that notwithstanding the extensive medical establishments of the India Company in the East, and the known ability which very generally characterises their officers there, we have scarcely any detached account of the climate and diseases of that vast empire, while volumes after volumes have issued from the press on the climate and diseases of the West India Islands." Preface, page 11. "Where, for instance, (says he) are we to look for any modern account of the endemic of Batavia, a settlement over which the British flag now waves, and whose very *name* is associated in the European mind with sickness and death! As for hepatitis, it is scarcely noticed by tropical writers, and we are forced to learn its nature and cure from a London physician who never set foot between the tropics." Ibid.

Mr. Johnson divides his work into three parts, each of which is subdivided into sections. Part 1,—Primary or general effects of a tropical climate on European constitutions, within the range of health. This contains four sections—on perspiration, sympathy, biliary secretion, and lichen tropicus.

The 2d part, on "Specific Diseases," is branched into eight sections on the following subjects. 1st. Endemic of Bengal, or marsh remittent fever. 2d. Bilious fever. 3d. Contagious fever. 4th. Endemic of Batavia. 5th. Endemic of the West Indies, or yellow fever. 6th. Hepatitis. 7th. Dysentery. 8th. Cholera morbus and Mort de Chien.

The 3d part, entitled "Prophylaxis, or Tropical Hygiene," contains eight sections also. 1st. On dress. 2d. On food. 3d. On drink. 4th. On exercise. 5th. On bathing. 6th. On sleep. 7th. On the passions. 8th. On naval hygiene and discipline.

In noticing the volume before us, we must confine our observations to a very few of the various subjects that occupy a closely printed octavo of more than 500 pages, referring to the work itself, which will probably obtain an extensive circulation.

In the 3d section of part 1, Mr. Johnson introduces a new principle, which lets in some light on tropical diseases, and is denominated by him the "*Cutaneo-hepatic sympathy.*" The *modus operandi* of heat, as a spur on the secretory vessels of the liver, has never been satisfactorily accounted for, and we think Mr. Johnson's elucidation will be considered as ingenious, and leading to practical advantages of considerable importance.

The arguments in support of his doctrine come in under hepatitis, dysentery, and other heads of the work. In this place he merely states the principle itself.

"There exists then between the extreme vessels of the vena portarum in the liver, and the extreme vessels on the surface of the body,—in other words, between *biliary secretion and perspiration,*—one of the strongest sympathies in the human frame, although entirely unnoticed hitherto, as far as I am acquainted. That these two functions are regularly, and, to appearance, equally increased, or at least influenced, by one particular agent (atmospheric heat) from the cradle to the grave, from the pole to the equator, will be readily granted by every observer; and that this *synchronous action* alone, independent of any other original connection, should soon grow up into a powerful sympathy, manifesting itself when either of these functions come under the influence of *other agents*, is a legitimate conclusion in theory, and what I hope to prove by a fair appeal to facts. But here I only offer assertions: in a future part of the work I shall bring forward facts and cogent arguments in proof of them. At present let this 'consent of parts' between the skin and liver, which I shall beg leave to denominate the *cutaneo-hepatic sympathy,* account for the augmented secretion of bile which we observe on arriving in hot climates, corresponding to the increased cuticular discharge." P. 20.

We shall have occasion to notice this sympathy hereafter.

The first section of part 2, on the Endemic of Bengal, opens opens with an animated sketch of the medical topography of that celebrated province, more especially the course of the Hoogly between Calcutta and Kedgeree, where Mr. J. informs us, on the authority of Captain Williamson, that full 300 European sailors annually fall victims to the marsh remittent fever! The fidelity of Dr. Clark's *description* of this endemic is contrasted with the inadequacy of that gentleman's *treatment*; and Mr. J. forcibly pourtrays the fatal consequences which must have resulted from the dread of *debility* and *putrescency* in fevers of the east; phantoms which, we have reason to know, still haunt the imaginations of numerous practitioners in that quarter of the globe, but which the present essay is well calculated to dispel. Mr. J.'s own plan of treatment, the result of painful experience, is thus described :—

"The first symptom that claims our most serious attention in this disease, is that irritability of the stomach, accompanied by a distressing vomiting. Till this is allayed, nothing can be done towards a cure by medicine. Now venesection has considerable effect in procuring alleviation even of this symptom, but the dribbling manner in which it is too often performed, when it is ventured on at all, does more harm than good. *Bleed boldly and decisively till the head and præcordia are relieved, or draw no blood whatever.* While this is doing, a scruple of calomel, with half a grain or a grain of opium, should be immediately given: this will act like a charm on the stomach. I shall prove, in the course of this essay, what indeed is well known to many of my brother officers who have served in India, that twenty grains of calomel will act as a *sedative;* and so far from griping, or producing hypercatharsis, it will soothe uneasiness, and rather constipate than purge. On this account, in the course of a few hours, when the vomiting is assuaged, some purgative must be given: cathartic extract with calomel, castor oil, or even salts, which will seldom fail to bring away a most copious discharge of intolerably fetid, bilious, and fæculent matter, to the unspeakable relief of the head and epigastrium. If there be now a return of any of those dangerous symptoms, intense head-ache, delirium, or pain in the epigastric region, no apprehension need be entertained of the lancet once more. Those bug-bears debility and putrescency still paralise the arms of medical men in hot climates, notwithstanding the clearest evidence in favor of venesection, particularly where the subject is lately from Europe, and not broken down by the climate. Immediately after the operation of the cathartic, the main-spring of the cure must be acted on. For this purpose, from five to ten grains of calomel, according to the urgency of the symptoms, combined with half a grain of opium, should be exhibited every four or six hours till ptyalism is raised, when, in nineteen cases out of twenty, there will be a remission of all the symptoms, and safety secured." P. 45-46.

Mr. J. condemns the use of emetics, and recommends cold cold applications to the head, with pediluvium, and a cathartic every day.

In a long and interesting dissertation on marsh miasmata, Mr. J. brings forward numerous original facts and curious incidents illustrative of the nature and effects of these invisible agents on the human constitution, accompanied with many judicious cautions in guarding against their deleterious influence.

In this section, Mr. Johnson has ventured, apparently with hesitation, into the intricate maze of theory. Aware of the difficulty, perhaps the inutility, of the subject, he confines himself to an attempt at explaining the more prominent features of the *ratio symptomatum* in fever; and, as there is both novelty and ingenuity in this part of the work, we shall allow Mr. J. to explain in his own words.

"First then, we need not be ashamed, however unfashionable (query does he not mean fashionable?) it is, to conclude with the immortal Cullen, that the remote cause of this fever, as well as that from contagion, is a sedative. That its application or reception diminishes the sensorial energy. That the power of the heart and arteries is first weakened, the consequence of which is an inability to propel the blood to the surface: hence the quiescence of the capillaries, the shrinking and coldness of the external parts, *without the intervention of spasm.* In this state, it follows of course, and is allowed by all, that the blood is confined to the heart and large internal trunks of vessels. But this does not appear sufficient to account for the swelling, tension, oppression, and even pain about the hypochondria, as well as many other of the symptoms attendant on the cold stage in particular. If, during the latter, I place my finger on the radial artery, and endeavour to estimate its calibre, and the quantum of blood transmitted through it, in a given time, compared with what takes place in the hot stage, or even in health, I shall conclude that the artery is not then above one third the size, nor the quantity of blood passing through it, more in proportion. Such being the case, it is difficult to conceive how the whole mass of blood can be in *actual circulation* at the time. In addition, therefore, to the confinement of a large share of it to the heart and great vessels, when its motion must be slow, I suppose another considerable portion of it to be *arrested,* as it were, and accumulated in certain situations, where it remains, *pro tempore, out of the course of the circulation.** This congestion or complete quiescence takes place in the *portal circle,* where the blood is at all times languid in its current, there being no *vis a tergo,* and but little muscular propulsion. The consequence of this must be, that not only the liver and the various branches of the vena portarum will become turgid, but also the spleen, (which

* He ought to have said, "out of the course of *actual* circulation."—*Rev.*

returns returns its blood to the heart through this channel) the stomach, pancreas, and intestines, will feel the effects of this turgescence. If it be asked why the blood should cease to circulate in these parts sooner than in others? I answer that the portal is the only circle or set of vessels in the sanguiferous system, *originating and terminating* in capillary tubes, or inosculations with other vessels. They begin by the minutest threads from the stomach, spleen, pancreas, and intestines; enlarge as they approach the liver, where they diverge, and finally dwindle again into the same diminutive size with which they commenced. All other veins dilate as they approximate to the heart, affording more and more facility to the return of the blood, which is in most places assisted by the action of circumjacent muscles. The temporary quiescence or torpor then, of the extreme branches of the vena portarum in the liver, from sympathy with the extreme vessels on the surface (before alluded to) must completely check and arrest the reflux of blood from the whole of the viscera above-mentioned. This state of things at once explains the tension, elevation, pain, weight, and anxiety about the præcordia. It shows why the biliary and pancreatic secretions are entirely suppressed for the time, while the gradual accumulation and temporary abstraction, as it were, of so great a proportion of the vital fluid from the circulation, will readily account for most if not all the phenomena of the cold stage, many of which were before inexplicable." P. 96.

Mr. J. pursues the subject with great ingenuity, but our limits prevent us from accompanying him. The next section, on bilious fever, presents us with a practical view of that disease as it exhibited itself on the great mass of a ship's company; and, although we cannot agree with Mr. Johnson in separating this from the marsh remittent fever, his practice and observations appear to be sound, and merit great attention. If this essay does not open the eyes of medical men towards the utility or rather the necessity of venesection and intestinal evacuations in fevers of the east, they must be proof against the clearest deductions.

The 4th section, on Endemic of Batavia, will be read with more than usual interest at the present time, as it is the only detailed or minute account which we have of that dreadful fever. The medical history of the expedition to that settlement is related with animation, and the cases are detailed with clearness and Hippocratic terseness. The pernicious effects of bark and wine are here placed in a conspicuous view, and the great determination to the liver and brain are fully demonstrated.

The 5th section, on Yellow Fever, or Endemic of the West Indies, written, as Mr. Johnson informs us, by a physician of first-rate talents, now in this country, and who had charge of an hospital nearly six years in the West Indies, is a most valuable communication, and, we trust, will be instrumental in saving the lives of thousands of our countrymen who come within the range of that fatal endemic. Its author disclaims the idea of contagion, and details with singular perspicuity the means of subduing this herculean fever by venesection, cold affusion, mercurial purgatives, and, as the vascular action subsides, the gradual exhibition of wine or other stimulants, particularly the carbonate of ammonia. From this last medicine being so strongly recommended, we suspect the present physician of Deal Hospital to be the ingenious author of the section in question, though for the truth of this we do not pledge ourselves.

In the 6th section, on Hepatitis, the influence of hot climates on Europ̀ean constitutions is placed in a newer and clearer light than in any work with which we are acquainted. It is here that Mr. J. brings forward numerous cogent proofs of the sympathetic connection between the skin and liver, or perspiration and biliary secretion, as detailed from page 265 to page 275. This subject is calculated to excite a more than usual interest in the medical world. After a full detail of the symptoms and treatment, a dissertation follows on the reciprocal influence which the mental and hepatic functions exert on each other; and this part of the work, though not strictly of a practical nature, gives us a very favorable idea of the author's reasoning powers.

The 7th section, on Dysentery, opens with a biting irony on Dr. Moseley's extravagant predilection for the ancients and their prescriptions in this disease. The discrepancy of opinion, and contrariety of practice, in dysentery, are strikingly set forth as puzzling the young beginner more than even fever. Mr. Johnson appears particularly happy in his application of that principle or sympathy (cutaneo-hepatic) of which we have spoken, both to the proximate cause and cure of this formidable complaint. We shall here introduce a short extract to convey his meaning in his own words.

"In every case of dysentery that has ever come within the range of my observation, (and the number has not been inconsiderable) two functions were invariably disordered from their very onset, and soon drew other derangements in their train. These were the functions of the skin and of the liver, or perspiration and biliary secretion. I defy any one, who has minutely regarded the disease, at the bed-side, to produce a single instance in which these functions were carried on in a natural manner, at any period of the disease." P. 354.

"These, then, are the two first links of that morbid chain connecting the the remote cause with the ostensible form of the disease. Whoever can break these by restoring those two functions to their natural state, I care not by what means or medicines, he will cure, or rather prevent, the disease—'et erit mihi magnus Apollo.' Some other invisible, at least very obscure links are now to be noticed, for, however confidently a *proximate* cause may be decided on in colleges and closets, it is in nature a series of causes. The equilibrium of the circulation becomes disturbed. In consequence of the torpor in the extreme vessels on the surface, the volume of blood is directed to the interior, and the balance is still farther broken by the check which the portal current meets in the liver, from a corresponding torpor in the extreme or secreting vessels of that organ, the effect of which is that the plethora in the cœliac and mesenteric circles is now greatly augmented, and febrile symptoms commence. The perspiration being stopped, a vicarious discharge of mucus and acrid serum is thrown from the extremities of the turgid mesenteric vessels upon the internal surface of the intestines, which, by this time, are in a state of irritability." P. 356.

But we cannot pursue this subject further, nor analyse the succeeding sections, all of which are exceedingly interesting. We were about to make some reflections and pass some censures on the severity which Mr. J. occasionally evinces to his contemporaries, but we have found so much to admire that we are unwilling to exercise our critical functions in finding fault. L.

